# Methods to estimate underlying blood pressure: The Atherosclerosis Risk in Communities (ARIC) Study

**DOI:** 10.1371/journal.pone.0179234

**Published:** 2017-07-11

**Authors:** Poojitha Balakrishnan, Terri Beaty, J. Hunter Young, Elizabeth Colantuoni, Kunihiro Matsushita

**Affiliations:** 1 Department of Environmental Health Sciences, Columbia University School of Public Health, New York, New York, United States of America; 2 Department of Epidemiology, Johns Hopkins School of Public Health, Baltimore, Maryland, United States of America; 3 Department of Biostatistics, Johns Hopkins School of Public Health, Baltimore, Maryland, United States of America; 4 Welch Center for Prevention, Epidemiology and Clinical Research, Johns Hopkins Medical Institutions, Baltimore, Maryland, United States of America; 5 Department of Medicine, Johns Hopkins School of Medicine, Baltimore, Maryland, United States of America; Shanghai Institute of Hypertension, CHINA

## Abstract

Antihypertensive medications complicate studies of blood pressure (BP) natural history; BP if untreated (“underlying BP”) needs to be estimated. Our objectives were to compare validity of five missing data imputation methods to estimate underlying BP and longitudinal associations of underlying BP and age. We simulated BP treatment in untreated hypertensive participants from Atherosclerosis Risk in Communities (ARIC) in visits 1–5 (1987–2013) using matched treated hypertensive participants. The underlying BP was imputed: #1, set as missing; #2, add 10 mmHg for systolic, 5 mmHg for diastolic; #3, add medication class-specific constant; #4, truncated normal regression; and #5, truncated normal regression including prior visit data. Longitudinal associations were estimated using linear mixed models of imputed underlying BP for simulated treated and measured BP for untreated participants. Method 3 was the best-performing for systolic BP; lowest relative bias (5.3% for intercept at age 50, 0% for age coefficient) and average deviation from expected (0.04 to -1.79). Method 2 performed best for diastolic BP; lowest relative bias (0.6% intercept at age 50, 33.3% age <60, 9.1% age 60+) and average deviation (-1.25 to -1.68). Methods 4 and 5 were comparable or slightly inferior. In conclusion, constant addition methods yielded valid and precise underlying BP and longitudinal associations.

## Introduction

Hypertension, systolic blood pressure (SBP) ≥140 mmHg or diastolic blood pressure (DBP) ≥90 mmHg, is prevalent in the U.S, affecting more than 30% of adults [1–3]. The incidence of hypertension is rising with lifetime estimates approaching 90% [4,5]. Along with increasing prevalence, antihypertensive medication use is growing [6,7]. This trend is in part also attributed to the increase in hypertension awareness [6,7]. Among U.S. adults with hypertension, the prevalence of antihypertensive medication use has increased from 63.5% in 2001–2 to 77.3% in 2009–10 [6].

Increasing use of antihypertensive medications is certainly important for preventing complications of hypertension but makes studies of blood pressure (BP) natural history difficult to investigate. For instance, setting BP to missing for all subjects receiving antihypertensive treatment can substantially reduce the sample size and power to detect effects of interest. Alternatively, including treated BP measures while investigating BP natural history can bias results towards subset of non-hypertensive subjects. In order to prevent these biases, several methods have been proposed to impute the “underlying BP”, BP that would have been measured if the individual was not taking antihypertensive medications. Adding a constant BP to the measured BP of the treated individual has been most commonly used, especially in genetic epidemiology [8–10]. More recent methodology incorporates regression-based imputation [8,11,12]. However most of these studies focus on evaluating in cross-sectionally [8,11], and few have investigated longitudinal settings [12], a time frame more relevant to natural history. Indeed, multiple BP measurements in longitudinal studies may amplify biases of not accounting for treatment. Alternatively, prior BP under no antihypertensive medications in longitudinal settings may be informative to estimate underlying BP in case an individual subsequently starts taking antihypertensive medications.

Therefore, the objective of this study is to comprehensively compare existing methods to impute underlying BP among individuals taking antihypertensive medications in a longitudinal setting using data from a large community-based cohort, the Atherosclerosis Risk in Communities (ARIC) Study, with repeated BP measurements over 26 years of follow-up.

## Materials and methods

### Study population

The ARIC Study recruited 15,792 individuals aged 45–64 years from four communities in Forsyth County, NC; Jackson, MS; northwest suburbs of Minneapolis, MN; and Washington County, MD. To date, ARIC has completed the baseline in 1987–89 and four follow-up visits in 1990–92, 1993–95, 1996–98 and 2011–13, respectively. Detailed study design and methods have been previously described [13,14]. All visits included interviews, anthropometric measurements, and BP measurements under a common protocol.

BP was measured via a random zero sphygmomanometer in visits 1–4 and an automatic sphygmomanometer (OMRON HEM-907 XL) in visit 5 by a certified trained technician in accordance with ARIC manual of procedures [13,15]. Briefly, participants were asked not to smoke, eat, perform physical exertion or experience cold temperatures 30 minutes prior to measurements. Participants were also asked to stay in a sitting position for at least 5 minutes prior to measurements. Each participant contributed three BP measurements per visit for visits 1–3 and 5 and the reported BP was an average of the second and third measurements [13–15]. For visit 4, BP was measured twice and reported as the average [13,14].

For this study, participants with measurements of SBP and DBP at 2 or more visits and data on antihypertensive medication use and class of medication were included. Other requisite variables were age, sex, race, and body mass index (BMI). Detailed inclusion criteria for visits 1–5 are presented in S2 Table. The ARIC study has been approved by Institutional Review Boards (IRB) at all participating institutions: University of North Carolina at Chapel Hill IRB, Johns Hopkins University IRB, University of Minnesota IRB, and University of Mississippi Medical Center IRB. Study participants provided written informed consent at all study visits.

### Simulation study

We generated hypothetical studies where the goal was to evaluate the natural history of underlying BP but where some patients were treated for hypertension. The hypothetical study population consisted of all non-hypertensive participants (SBP <140 mmHg, DBP <90 mmHg, not taking antihypertensive medications) and untreated hypertensive participants (not taking antihypertensive medications despite either SBP ≥140 mmHg or DBP ≥90 mmHg) at each visit. We will refer to the BP among the untreated hypertensive participants as “measured untreated BP”. Then, we simulated treatment for the untreated hypertensive participants by assigning a hypothetical treated BP (“simulated treated BP”) as the measured BP from a matched treated hypertensive participant (taking antihypertensive medications). Matching was performed using strong predictors of BP, including age ± 5 years, sex, race and BMI ± 5 kg/m^2^. Using antihypertensive medication literature to guide us [6–8,10,16,17], we also included in the matching criteria that the simulated treated BP to be within a conservative interval of 20 mmHg for SBP and 10 mmHg for DBP of the measured untreated BP. If more than one treated hypertensive subject satisfied the above matching criteria, then one of the subjects that met the criteria was chosen at random.

We subsequently applied five methods to account for the treatment of the hypertensive patients within our hypothetical study. First, we set the underlying BP value to missing for all participants in the hypothetical study that were taking antihypertensive medications (Method 1). The remaining four methods imputed the underlying BP using the simulated treated BP. Method 2 (simple constant addition) added a constant of 10 mmHg to the simulated treated SBP and 5 mmHg to the simulated treated DBP [8,10,12] and method 3 (medication class-specific constant addition) was to add a constant to the simulated treated SBP and DBP depending on the individuals’ medication class. The constants were derived from a summary report of the effects of antihypertensive medication classes [16]. For six representative medication classes [3,16], we applied the following expected treatment effect in mmHg for SBP/DBP as constants: 14.3/10.4 if European American and 6.8/6.6 if African Americans for angiotensin-converting enzyme (ACE) inhibitors, 15.5/11.7 for alpha blockers, 14.8/12.2 for beta blockers, 15.3/10.5 for calcium channel blockers (including dihydropyridine and non- dihydropyridine), 15.5/9.0 for diuretics (including thiazide-like and loop), and 14.8/10.5 for miscellaneous BP-lowering medications [16]. Race specific medication constants were used for ACE inhibitors due to the clear difference by race unlike the other five medication classes (S1 Table) [16]. If the participant was taking combination therapy, the medication with the largest expected monotherapy effect was chosen as the primary medication and the non-primary medications had a percentage of their monotherapy effect listed above [16]. Detailed description of the estimated treatment effects is provided elsewhere [16], and a summary can be found in S1 Table. For example, if a participant was taking diuretics and beta blockers, the total expected effect is 17.1 mmHg SBP/13.1 mmHg DBP; the primary medication effect for diuretics is 15.5 mmHg SBP/9.0 mmHg DBP and the non-primary medication effect for beta-blockers is 1.6 mmHg SBP (14.8x11%)/4.1 mmHg DBP (12.2x34%).

Methods 4 and 5 imputed the underlying BP using a regression-based approach. Method 4 (truncated normal regression) used a censored normal distribution to generate the imputed underlying BP by assuming that the distribution of underlying BP is normally distributed and that the underlying BP is higher than the measured BP under treatment [8]. The censored normal model was fit using *a priori* selected predictors of underlying BP from previous literature [1–3] and empirically included if P<0.05. The SBP model included untreated hypertensive participants’ age, age^2^, race, sex, BMI, height, height^2^, and interaction terms for age and age^2^ with the sex and height variables. The DBP model included the above variables and instead of age^2^, a spline term for age at 60 years and its respective interaction terms with sex, height, and height^2^. Detailed methodology of censored normal regression has been previously published [8]. Briefly, the underlying BP for a treated participant was imputed by randomly sampling from a normal distribution with mean defined as the model predicted mean and standard deviation defined as the model mean squared error where the imputed underlying BP was greater than the simulated treated BP. This random sampling was repeated 15 times and the final imputed underlying BP was an average of the 15 values in order to incorporate variability. Method 5 (truncated normal regression with prior visit BP and antihypertensive treatment) was the same as method 4 but included two extra predictors, measured BP and the use of antihypertensive medications at the prior visit, in the censored normal regression model.

We simulated 1000 hypothetical studies and applied each of the five methods above to each simulated study (S1 Fig). As sensitivity analyses, we modified methods 4 and 5 and used 1 instead of 15 repeated sampling of matching in order to incorporate BP variability. All analysis was performed using R version 3.2.0 [18].

### Validity of imputation methods

The validity of the imputation methods was assessed several ways. First, we compared the distribution of the imputed underlying BP based on methods 2 through 5 to the measured untreated BP. The average difference between the measured untreated BP and the imputed underlying BP was calculated for each visit. In addition, we assessed the difference in the empirical distributions for each of the 1000 simulations using the Anderson-Darling test. The utility of the Anderson-Darling test may be limited in conditions with large sample size and deviances from a normal distribution. We interpreted our results within the context of these limitations.

Subsequently, we obtained the true association between underlying BP and age using the measured BP values for the hypothetical study population; all non-hypertensive participants (SBP <140 mmHg, DBP <90 mmHg, not taking antihypertensive medications) and untreated hypertensive participants; by fitting a linear mixed model including fixed effects for age (spline at 60 years for DBP, as aforementioned) and random effects at the patient level for both the intercept (centered at 50 years) and age term(s). The same linear mixed models were fit to the 1000 hypothetical studies applying methods 1 through 5. The relative bias of each method was defined as the relative difference in the average regression coefficient compared to the true regression coefficient and was calculated for the model intercept and age term(s).

Our *a priori* hypothesis was that method 5 would outperform the other methods because the method was regression-based and incorporated the tracking of prior BP. All analysis was performed using R version 3.2.0 [18].

## Results

### Matching and simulation

In brief, 10,283 participants were included in the hypothetical study population, of whom at visit 1, 9,122 were non-hypertensive and 1,161 were untreated hypertensive and were assigned simulated treatment. Detailed exclusion criteria (S2 Table) and demographic characteristics of participants at all other visits (S3–S6 Tables) are provided as supporting information. Results are primarily presented below using visit 2 since visit 1 was not informative for imputation methods using prior visit data (method 5) and since results were consistent across the remaining visits with the largest sample size at visit 2.

**Fig 1:**
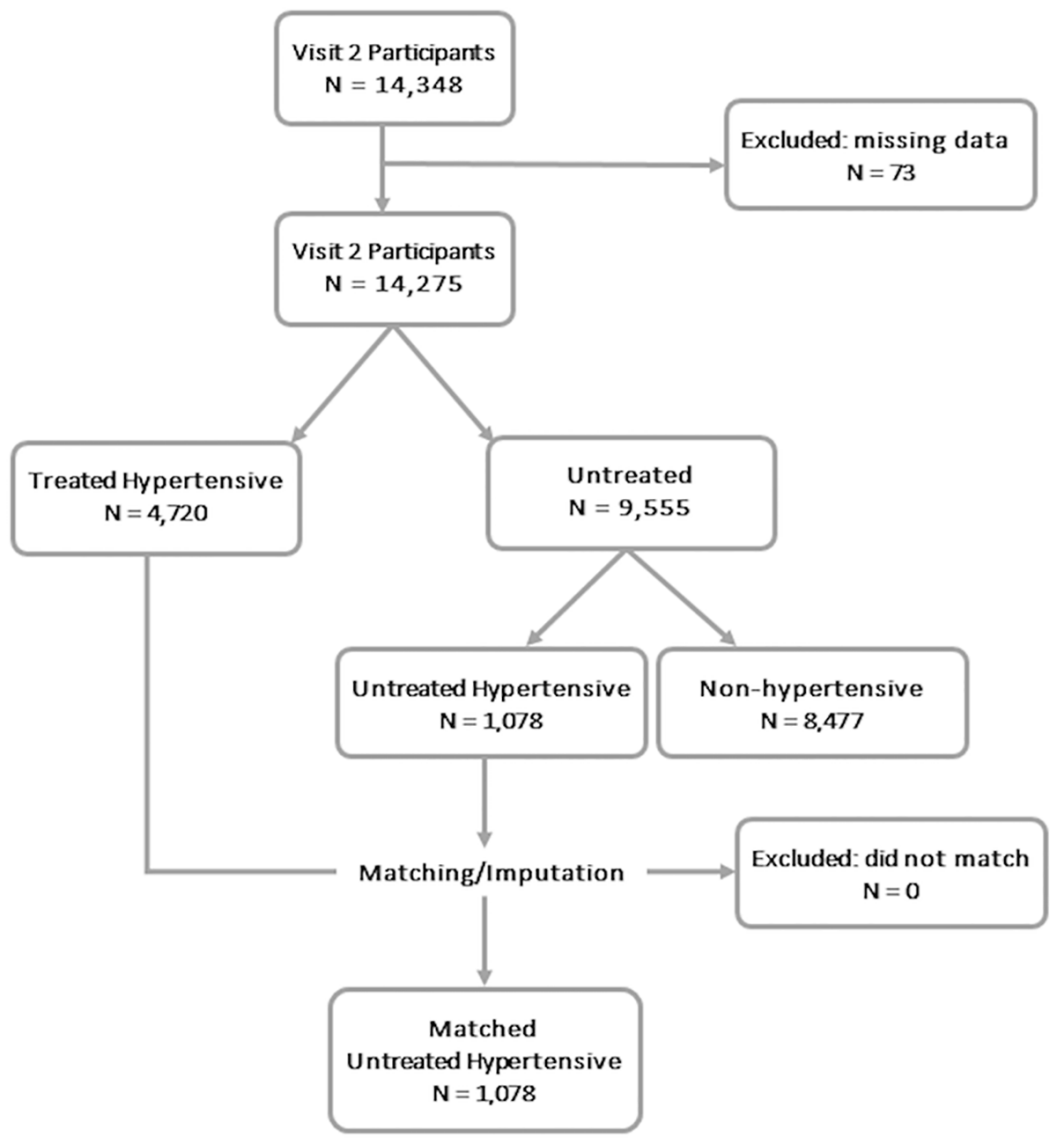
Matching and imputation scheme for visit 2. At each visit, 14,275 out of 14,348 participants were included after exclusion for missing age, sex, race, body mass index, antihypertensive medication use and antihypertensive medication class as well as if they had only 1 visit’s blood pressure (BP). Among the 14,275 participants without missing data, 9,555 participants were not taking antihypertensive medications. 1,078 of these participants were not taking antihypertensive medications with elevated BP. These untreated hypertensive participants were matched to one of the treated hypertensive participant (N = 4,720) to simulated the treated BP.

A total of 1,078 untreated hypertensive participants and 4,720 treated hypertensive participants had complete data at visit 2 (Fig 1). Table 1 shows the demographic and clinical characteristics of 1,078 untreated hypertensive participants compared to the 4,720 treated hypertensive participants at visit 2. The untreated and treated hypertensive participants were of comparable age and BMI. In general, the untreated hypertensive participants were more likely to be male, White and educated at least high school level. Compared to the treated hypertensive participants, comorbid conditions were less prevalent in untreated hypertensive participants, including kidney dysfunction (estimated glomerular filtration rate [eGFR] <60 ml/min/1.73m^2^), diabetes, prevalent coronary heart disease (CHD), parental history of CHD, and history of heart failure. All of the untreated hypertensive participants were matched to a treated hypertensive participant according to the criteria previously described (Fig 1). The distribution of simulated treated BP was shifted towards lower BP compared to the distribution of measured untreated BP, as expected (Table 2).

**Table 1:** Summary of participant characteristics at visit 2.

	Untreated Hypertensive Participants (N = 1,078)	Treated Hypertensive Participants (N = 4,720)	P-value
Mean age, yrs (SD)	58.1 (5.8)	58.3 (5.7)	0.47
Male (%)	540 (50.1)	1992 (42.2)	<0.01
Race (% Blacks)	333 (30.9)	1697 (36.0)	<0.01
Mean BMI*, kg/m^2^ (SD)	28.7 (5.9)	29.7 (6.0)	<0.01
Center (%)			<0.01
Forsythe	223 (20.7)	1014 (21.5)	
Jackson	290 (26.9)	1500 (31.8)	
Minneapolis	327 (30.3)	957 (20.3)	
Washington	238 (22.1)	1249 (26.5)	
Education less than high school (%)	812 (75.4)	3307 (70.2)	<0.01
Current smokers (%)	235 (21.8)	965 (20.5)	0.35
Current drinkers (%)	628 (58.4)	2230 (47.4)	<0.01
Kidney dysfunction (%)	20 (1.9)	238 (5.1)	<0.01
Diabetes (%)	161 (15.0)	1198 (25.6)	<0.01
Prevalent CHD (%)	31 (3.0)	584 (12.6)	<0.01
Prevalent heart failure (%)	26 (2.5)	520 (11.2)	<0.01
Parental history of CHD* (%)	75 (8.4)	472 (12.4)	<0.01

Out of a total of 10,283 participants, untreated hypertensive participants who went through simulation and imputation at visits 1–5 were 1,161, 1,078, 1,012, 1,025, and 333, respectively. Abbreviations: BMI, body mass index; CHD, coronary heart disease; SD, standard deviation.

**Table 2:** Accuracy of imputed underlying BP to measured untreated BP.

	Method 2			Method 3			Method 4			Method 5		
	Diff	A-D	P-value	Diff	A-D	P-value	Diff	A-D	P-value	Diff	A-D	P-value
**SBP**												
Visit 1	5.11	60.70	1.79e-33	0.04	24.80	7.80e-14	6.51	76.70	2.80e-42	6.51	76.70	2.80e-42
Visit 2	4.96	59.20	1.13e-32	0.89	35.70	8.09e-20	6.46	70.90	4.23e-39	5.09	53.10	2.51e-29
Visit 3	5.23	72.80	4.00e-40	1.25	42.80	1.11e-23	6.23	76.90	2.35e-42	4.56	52.70	3.95e-29
Visit 4	4.48	59.50	7.88e-33	-0.19	58.20	3.83e-32	4.95	55.60	1.10e-30	3.54	55.20	1.30e-30
Visit 5	3.91	21.40	5.60e-12	-1.79	16.40	2.92e-9	3.03	14.50	3.32e-8	2.95	12.70	3.36e-7
**DBP**												
Visit 1	-1.25	10.90	3.18e-6	-7.14	109.00	3.14e-60	-1.77	19.90	4.02e-11	-1.77	19.90	4.02e-11
Visit 2	-1.57	11.30	2.04e-6	-6.81	95.70	1.16e-52	-1.93	19.90	3.91e-11	-1.64	12.10	7.26e-7
Visit 3	-1.68	9.21	2.73e-5	-6.99	85.90	2.55e-47	-2.75	26.80	6.26e-15	-2.44	17.20	1.10e-9
Visit 4	-1.67	9.16	2.88e-5	-7.61	101.00	9.08e-56	-3.26	34.80	2.70e-19	-2.83	19.80	4.14e-11
Visit 5	-1.67	2.99	0.03	-8.66	41.10	8.53e-23	-4.08	18.00	4.26e-10	-3.81	13.90	6.96e-8

The mean difference was calculated between the measured untreated BP and the imputed underlying BP. The resulting Anderson-Darling statistic and p-value estimates the accuracy of imputation methods 2–5. Abbreviations: Diff, difference; A-D, Anderson-Darling test statistic; SBP, systolic blood pressure; DBP, diastolic blood pressure.

### Imputed underlying BP

Table 2 shows the relative accuracy of methods 2 through 5 at imputing the underlying SBP and DBP (Table 2). In general, the best-performing method was the same for each visit for SBP and DBP, respectively. Method 3 (class-specific constant addition) was the most accurate for imputing underlying SBP with the mean difference between imputed underlying and underlying SBP ranging from 0.04 at visit 1 to -1.79 at visit 5. Methods 2, 4 and 5 had similar performance with the mean difference in imputed underlying and underlying SBP of roughly 5. For DBP, method 2 had the lowest mean difference in imputed underlying and underlying DBP across five visits (minimum of -1.25 at visit 1 and maximum of -1.68 at visit 3). After method 2, method 5 followed by methods 4 and 3 had the lowest mean difference between measured untreated and imputed underlying DBP. The distributions of the imputed underlying BP were significantly different than the distributions of the measured untreated BP (Table 2), although the impact of the large sample size on the statistical significance cannot be discounted.

**Fig 2:**
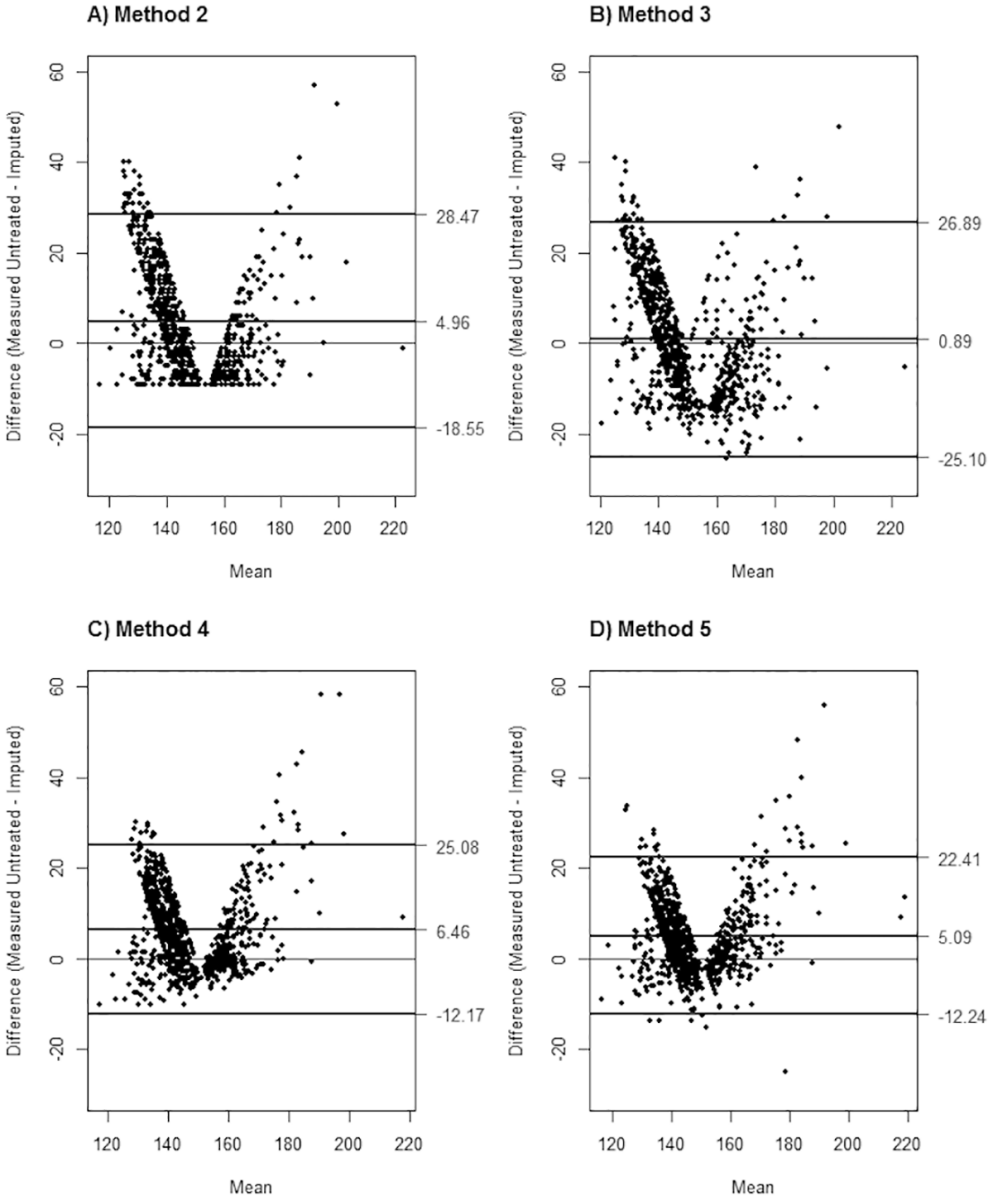
Bland-Altman plot of imputed underlying SBP at visit 2. The measured untreated systolic blood pressure (SBP) was treated as gold standard for the Bland-Altman plots with confidence intervals at 2.5%-97.5%. Imputed underlying SBP was derived using methods 2–5 (A-D).

While the mean difference between the imputed underlying and underlying BP gives an estimate of the average accuracy, the relative accuracy of the imputed underlying BP differed by the underlying BP. Using one round of SBP imputation at visit 2 as an example (Fig 2), the imputed underlying SBP tended to underestimate the underlying BP at the outer ranges of the measured untreated SBP, especially the upper ranges, resulting in V-shaped Bland Altman plots. This V-shaped pattern was apparent in methods 2–4. The V-shape was partly due to the matching criteria, which enforced a lower and upper bound in the simulated treated BP, and partly due to the applicability of each method’s assumptions at the extremes of the simulated treated BP. The precision of the estimation was also comparable across methods although the regression-based methods tended to be more precise as expected (Fig 2). Although methods 2–5 demonstrated wide 95% limits of agreement (34.65 to 51.99 mmHg), method 5 (truncated normal regression with prior visit BP and antihypertensive treatment) yielded the most precise estimation followed by method 4 (truncated normal regression). The pattern observed for this round of imputation for visit 2 was also noted in visits 3–5 and in other rounds of imputation for both SBP and DBP (S2–S6 Tables).

### Longitudinal associations of imputed underlying BP with age

The results of the longitudinal analysis are presented in Table 3 for SBP and DBP. Although estimations of intercept and age are important for validity, we highlighted the age associations given the longitudinal nature of our study. The linear association between underlying SBP and age was underestimated (by 28%) using method 1, where we set the underlying SPB missing for patients who were treated for hypertension. In comparison, methods 2–4 tended to have little to no bias (<6%), with lowest bias for method 3. For DBP, the coefficients measuring the true association between underlying DBP and age were small (0.03 and -0.11 before and after age 60 years, respectively) and thus even small differences in the average age coefficients across the imputation methods translated to larger relative bias compared to the SBP analysis. Nevertheless, method 1 yielded the highest bias (200% and 72.7%, respectively). Method 2 yielded the least biased estimates for both coefficients for age (relative bias 33.3% and 9.1%, respectively). Method 5 also yielded relatively little bias (relative bias 33.3% and 45.5%, respectively).

**Table 3:** Bias in intercept and age associations from linear mixed effects models.

		Measured	Method 1	Method 2	Method 3	Method 4	Method 5
**SBP**							
Intercept	Beta (Var)	114.20	116.95	120.60 (5.55e-4)	120.28 (8.25e-4)	120.48 (4.29e-4)	120.62 (3.43e-4)
	Relative bias		2.4	5.6	5.3	5.5	5.6
Age	Beta (Var)	0.90	0.65	0.85 (8.87e-6)	0.90 (1.36e-5)	0.87 (6.87e-6)	0.88 (6.31e-6)
	Relative bias		27.8	5.6	0.0	3.3	2.2
**DBP**							
Intercept	Beta (Var)	71.41	69.97	71.85 (7.05e-4)	68.30 (1.52e-2)	71.83 (5.18e-4)	71.79 (1.53e-4)
	Relative bias		2.0	0.6	4.4	0.6	0.5
Age before 60 yrs	Beta (Var)	0.03	-0.03	0.04 (1.89e-6)	0.07 (3.93e-6)	0.05 (1.00e-6)	0.04 (1.28e-6)
	Relative bias		200.0	33.3	133.3	66.7	33.3
Age after 60 yrs	Beta (Var)	-0.11	-0.19	-0.10 (7.16e-6)	-0.03 (3.39e-5)	-0.05 (7.16e-6)	-0.06 (3.80e-6)
	Relative bias		72.7	9.1	72.7	54.6	45.5

The relative bias (%) was calculated as the average relative difference in regression coefficient from each simulation (100 x [β_imputation method_−β_measured untreated BP_] / β_measured untreated BP_). Abbreviations: SBP, systolic blood pressure; Var, variance; DBP, diastolic blood pressure.

## Discussion

This longitudinal simulation study demonstrated the need for imputation methods to account for the effect of antihypertensive medications, especially in cohorts drawn from the general population where medication use is common. As anticipated, setting the treated BP measurements as missing was especially biased. In addition, constant addition methods (methods 2 and 3) tended to be superior to the other methods in both estimating underlying BP and in the longitudinal analysis of underlying BP and its change over age. Although the truncated normal regression methods (methods 4 and 5) were comparable, unexpectedly they did not outperform methods 2 and 3 overall. Therefore, a hybrid of the method 3 for SBP and method 2 for DBP may be an option for epidemiological studies focusing on both SBP and DBP.

Our findings are consistent with previous cross-sectional literature. Tobin et al. reported that the constant addition is better than or equivalent to the truncated normal regression in cross-sectional setting [8] and surprisingly, we confirmed this in a longitudinal setting. There may be several reasons behind the validity of constant addition methods. Firstly since the definition and management of hypertension are well established, treatment itself and the medication classes may be more relevant to estimating underlying BP than individual characteristics such as age and sex. Second, previous work to account for medication use addressed the issue of underlying BP as a missing data problem [8,11,12,19]. In theory, imputation methods such as truncated normal regression that are based on variables in the dataset assume that the dependent variable (i.e. underlying BP) is missing at random (MAR) [8,11,12,19]. However since underlying BP can be missing not at random (MNAR) or can be due to variables that were unobserved, not measured well or not included in the model, regression based methods may not perform as well as expected [8,11,12,19]. This could also contribute to why ignoring the missingness of underlying BP (method 1) would not be appropriate, as reported previously [8,11,12,19]. For other traits less variable over time such as cholesterol, regression-based methods may outperform constant addition methods [20,21]. This may even be true for BP if measured more often. Nevertheless, constant addition methods are simpler compared to truncated normal regression and therefore advantageous in the context of large-scale epidemiological investigations.

Among constant methods, method 3 for SBP and method 2 for DBP performed well. The discrepancy could be explained in part due to how DBP vs. SBP responds to medication. In general SBP is well studied since it is a more established risk factor of future cardiovascular disease and other complications [2,3]. Thus, medications may be investigated for their effect on SBP more than their effect on DBP. Also just as the relationship between age and DBP is more complex [2], the relationship between DBP and medication class may be more difficult to summarize as a series of constants. In addition, the range of DBP is smaller than SBP and thus even slight measurement error in the studies measuring medication effects can be more influential for DBP. It is also important to note the large relative bias estimates for DBP is in part due to the small coefficients in the linear mixed models. Finally, we cannot discount that DBP compared to SBP may be less influenced by the medication class or combination of medications used for treatment. Thus adding 5mmHg may better capture the effects of antihypertensive medication use for DBP.

This is the first study to our knowledge to comprehensively assess the existing imputation methods of underlying BP in a longitudinal setting. While our imputation methods relied on previously published methods [8,12,22], we expanded on these methods by incorporating the heterogeneity of medication classes in method 3 and the tracking of BP over time in method 5. Also unlike previous studies, we used data from an existing cohort rather than simulate data based on a known model [8,12,22]. This allowed for a more empiric assessment of the imputation methods. Our empiric dataset was robust for addressing antihypertensive use; it was derived from a large, multi-center cohort with a large proportion of treated and untreated hypertensive participants. The ARIC cohort also included standard and reliable measurement of BP and covariates over nearly three decades.

There were some limitations to our study. Firstly, since the number of matches considerably decreased, we did not take into account comorbid conditions such as kidney dysfunction and diabetes during matching despite these altering the indication and the effects of antihypertensive medications. For instance, the guidelines for BP target levels are different among individuals with and without comorbidities such as diabetes. Among individuals with diabetes, the SBP target levels are <130–140 mmHg and the DBP target levels are <80 mmHg [23]. Nonetheless, the relative performance of the imputation methods was consistent with the main findings even when we included kidney function and diabetes in the matching criteria as a sensitivity analysis (S7 Table). Secondly, our assessment of BP relied on an average of two measurements on a single day in several years, although in clinical practice hypertension would be determined based on BP measurements at multiple occasions over several months. Thirdly, a constant threshold (SBP ≥140 mmHg or DBP ≥90 mmHg) was used in all five ARIC visits for the simulation even though ARIC visits occurred from 1987–2013 and thresholds for diagnosing and treating hypertension changed overtime (i.e., 160 mmHg SBP/90 mmHg DBP before 1992–3 and 140/90 mmHg since then) [24,25]. Since a constant cutoff over all visits irrespective of comorbidities was used, bias may have been introduced in classifying participants as untreated hypertensive and in the matching process. Finally, the adherence and dose of antihypertensive medications were not measured. Both factors could lead to heterogeneity in the effect of antihypertensive medications on BP and could affect the observed longitudinal associations.

## Conclusions

In summary, our longitudinal simulation study demonstrated that imputation methods can account for treatment in large-scale epidemiological studies of BP natural history. Although both constant addition methods and regression methods are useful, constant addition methods may outperform regression methods in estimating and modeling underlying BP, especially SBP. Further studies are required to assess generalizability and robustness of our findings accounting for possible modifiers of underlying BP such as dose, adherence, and cause of combination therapy.

## Supporting information

S1 Fig Summary of simulation study: Example participant. The figure illustrates an example participant (‘Participant A’) with elevated blood pressure (BP) who does not take antihypertensive medications (untreated hypertensive) and thus was eligible for the simulation study. Participant A’s BP (systolic blood pressure 153/diastolic blood pressure 93) was measured without antihypertensive treatment (measured untreated BP). Participant A was matched to a treated hypertensive participant with similar age, sex, race and body mass index (Participant X). Participant X’s BP (150/90) was used as the simulated treated BP (BP that would have been measured for Participant A had he/she been taking antihypertensive medications). Using the simulated treated BP, the following imputation methods were conducted: #1, set as missing; #2, single constant addition (systolic BP 10/diastolic BP 5); #3, class-specific constant addition; #4, truncated normal regression; #5, truncated normal regression with prior visit BP and antihypertensive treatment.
(TIF)

S2 Fig Bland-Altman plot of imputed underlying DBP at visit 2. The measured untreated diastolic blood pressure (DBP) was treated as gold standard for the Bland-Altman plots with confidence intervals at 2.5%-97.5%. Imputed underlying DBP was derived using methods 2–5 (A-D).
(TIF)

S1 Table Expected effects of antihypertensive therapy for sitting BP. Adapted from Wu J, Kraja AT, Oberman A, Lewis CE, Ellison RC, Arnett DK, et al. A summary of the effects of antihypertensive medications on measured blood pressure. Am J Hypertens 2005 Jul;18(7):935–942. The expected effect of each medication class was estimated and listed overall and by race for ACE inhibitors. If the medication was used as part of combination therapy, the average effects of the non-primary medication were reduced. This weighted effect was expected to be influenced by whether a diuretic was part of the combination therapy. Abbreviations: BP, blood pressure; SBP, systolic blood pressure; DBP, diastolic blood pressure; ACE, angiotensin-converting enzyme.
(DOCX)

S2 Table Summary of participants and inclusion criteria at visits 1–5. At each visit, participants were included in the analysis if they did not have missing age, sex, race, body mass index (BMI), antihypertensive medication use and antihypertensive medication class. Participants were further excluded if they had blood pressure (BP) measured at only 1 visit. Of the remaining participants, they were classified according to medication status (treated hypertensive, untreated) and BP levels among untreated (non-hypertensive, untreated hypertensive). Untreated hypertensive participants were included in the simulation study if they had at least 1 match among the treated hypertensive according to age, sex, race and BMI.
(DOCX)

S3 Table Summary of participant characteristics at visit 1. Abbreviations: BMI, body mass index; CHD, coronary heart disease; SD, standard deviation.
(DOCX)

S4 Table Summary of participant characteristics at visit 3. Abbreviations: BMI, body mass index; CHD, coronary heart disease; SD, standard deviation.
(DOCX)

S5 Table Summary of participant characteristics at visit 4. Abbreviations: BMI, body mass index; CHD, coronary heart disease; SD, standard deviation.
(DOCX)

S6 Table Summary of participant characteristics at visit 5. Abbreviations: BMI, body mass index; CHD, coronary heart disease; SD, standard deviation.
(DOCX)

S7 Table Accuracy of imputed underlying BP to measured untreated BP matched on diabetes and kidney function. The relative bias (%) was calculated as the average relative difference in regression coefficient from each simulation (100 x [β_imputation method_−β_measured untreated BP_] / β_measured untreated BP_). Controls were matched on diabetes and kidney dysfunction status. Abbreviations: SBP, systolic blood pressure; Var, variance; DBP, diastolic blood pressure.
(DOCX)

## References

[1] Centers for Disease Control and Prevention (CDC). Vital signs: awareness and treatment of uncontrolled hypertension among adults—United States, 2003–2010. MMWR Morb Mortal Wkly Rep 2012 9 7;61:703–709. 22951452

[2] Go AS, Mozaffarian D, Roger VL, Benjamin EJ, Berry JD, Blaha MJ, et al Heart disease and stroke statistics—2014 update: a report from the American Heart Association. Circulation 2014 1 21;129(3):e28–e292. doi: 10.1161/01.cir.0000441139.02102.80 24352519 10.1161/01.cir.0000441139.02102.80 PMC5408159

[3] Hindorff L, Junkins H, Hall P, Mehta J, Manolio T. A Catalog of Published Genome-Wide Association Studies. www.genome.gov/gwastudies. 2014.

[4] Vasan RS, Beiser A, Seshadri S, Larson MG, Kannel WB, D'Agostino RB, et al Residual lifetime risk for developing hypertension in middle-aged women and men: The Framingham Heart Study. JAMA 2002;287(8):1003–1010. 11866648 10.1001/jama.287.8.1003

[5] Hajjar I, Kotchen JM, Kotchen TA. Hypertension: trends in prevalence, incidence, and control. Annu Rev Public Health 2006;27:465–490. doi: 10.1146/annurev.publhealth.27.021405.102132 16533126 10.1146/annurev.publhealth.27.021405.102132

[6] Gu Q, Burt VL, Dillon CF, Yoon S. Trends in antihypertensive medication use and blood pressure control among United States adults with hypertension: the National Health And Nutrition Examination Survey, 2001 to 2010. Circulation 2012 10 23;126(17):2105–2114. doi: 10.1161/CIRCULATIONAHA.112.096156 23091084 10.1161/CIRCULATIONAHA.112.096156

[7] Centers for Disease Control and Prevention (CDC). Self-reported hypertension and use of antihypertensive medication among adults—United States, 2005–2009. MMWR Morb Mortal Wkly Rep 2013 4 5;62(13):237–244. 23552224 PMC4605009

[8] Tobin MD, Sheehan NA, Scurrah KJ, Burton PR. Adjusting for treatment effects in studies of quantitative traits: antihypertensive therapy and systolic blood pressure. Stat Med 2005;24(19):2911–2935. doi: 10.1002/sim.2165 16152135 10.1002/sim.2165

[9] Newton-Cheh C, Johnson T, Gateva V, Tobin MD, Bochud M, Coin L, et al Genome-wide association study identifies eight loci associated with blood pressure. Nat Genet 2009;41(6):666–676. doi: 10.1038/ng.361 19430483 10.1038/ng.361 PMC2891673

[10] Padmanabhan S, Newton-Cheh C, Dominiczak AF. Genetic basis of blood pressure and hypertension. Trends in Genetics 2012;28(8):397–408. doi: 10.1016/j.tig.2012.04.001 22622230 10.1016/j.tig.2012.04.001

[11] Kang T, Kraft P, Gauderman WJ, Thomas D, Framingham Heart Study. Multiple imputation methods for longitudinal blood pressure measurements from the Framingham Heart Study. BMC Genet 2003 12 31;4 Suppl 1:S43. 14975111 10.1186/1471-2156-4-S1-S43 PMC1866479

[12] Sun W, Larsen MD, Lachin JM. Methods for a longitudinal quantitative outcome with a multivariate Gaussian distribution multi-dimensionally censored by therapeutic intervention. Stat Med 2014;33(8):1288–1306. doi: 10.1002/sim.6037 24258796 10.1002/sim.6037

[13] ARIC Coordinating Center. Atherosclerosis Risk in Communities Cohort Procedures Visit 1. http://www2.cscc.unc.edu/aric/visit/Cohort_Procedures.1_2.pdf.

[14] ARIC investigators. The Atherosclerosis Risk in Communities (ARIC) Study: design and objectives. The ARIC investigators. Am J Epidemiol 1989 4;129(4):687–702. 2646917

[15] ARIC Coordinating Center. Home and Field Center Procedures ARIC Visit 5 and NCS Study Protocol. https://www2.cscc.unc.edu/aric/sites/default/files/public/manuals/Manual%202%20Home%20and%20Field%20Center%20Procedures.pdf.

[16] Wu J, Kraja AT, Oberman A, Lewis CE, Ellison RC, Arnett DK, et al A summary of the effects of antihypertensive medications on measured blood pressure. American journal of hypertension 2005;18(7):935–942. doi: 10.1016/j.amjhyper.2005.01.011 16053990 10.1016/j.amjhyper.2005.01.011

[17] Gu Q, Paulose-Ram R, Dillon C, Burt V. Antihypertensive medication use among US adults with hypertension. Circulation 2006 1 17;113(2):213–221. doi: 10.1161/CIRCULATIONAHA.105.542290 16391156 10.1161/CIRCULATIONAHA.105.542290

[18] R Core Team (2016). R: A language and environment for statistical computing. R Foundation for Statistical Computing, Vienna, Austria URL https://www.R-project.org/.

[19] Vaitsiakhovich T, Drichel D, Angisch M, Becker T, Herold C, Lacour A. Analysis of the progression of systolic blood pressure using imputation of missing phenotype values. BMC Proc 2014 6 17;8(Suppl 1):S83 6561-8-S1-S83. eCollection 2014. doi: 10.1186/1753-6561-8-S1-S83 25519344 10.1186/1753-6561-8-S1-S83 PMC4143701

[20] Jorgensen NW, Sibley CT, McClelland RL. Using imputed pre-treatment cholesterol in a propensity score model to reduce confounding by indication: results from the multi-ethnic study of atherosclerosis. BMC medical research methodology 2013;13(1):81. 23800038 10.1186/1471-2288-13-81 PMC3694006

[21] McClelland RL, Kronmal RA, Haessler J, Blumenthal RS, Goff DC. Estimation of risk factor associations when the response is influenced by medication use: an imputation approach. Stat Med 2008;27(24):5039–5053. doi: 10.1002/sim.3341 18613245 10.1002/sim.3341 PMC2829271

[22] Cook NR. An imputation method for non-ignorable missing data in studies of blood pressure. Stat Med 1997;16(23):2713–2728. 9421871 10.1002/(sici)1097-0258(19971215)16:23<2713::aid-sim705>3.0.co;2-s

[23] American Diabetes Association. Standards of medical care in diabetes—2014. Diabetes Care 2014 1;37 Suppl 1:S14–80. 24357209 10.2337/dc14-S014

[24] Frohlich ED. Detection, evaluation, and treatment of hypertension: JNC-5 (Joint National Committee on Detection, Evaluation, and Treatment of High Blood Pressure). Heart Dis Stroke 1993 Nov-Dec;2(6):459–460. 8137049

[25] Pogue VA, Ellis C, Michel J, Francis CK. New staging system of the fifth Joint National Committee report on the detection, evaluation, and treatment of high blood pressure (JNC-V) alters assessment of the severity and treatment of hypertension. Hypertension 1996 11;28(5):713–718. 8901813 10.1161/01.hyp.28.5.713

