# Supplementary figures and images for: Methods to estimate underlying blood pressure: The Atherosclerosis Risk in Communities (ARIC) Study
Source: PLoS One. 2017 Jul 11;12(7):e0179234. doi: 10.1371/journal.pone.0179234 (PMC5507409; doi:10.1371/journal.pone.0179234)

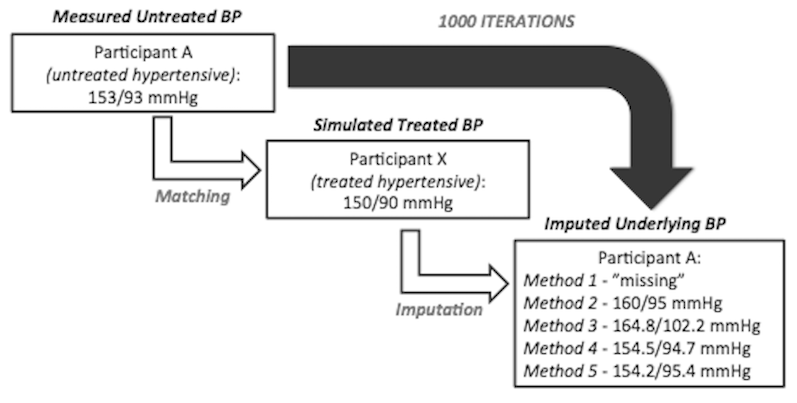

Supplement: S1 Fig — The figure illustrates an example participant (‘Participant A’) with elevated blood pressure (BP) who does not take antihypertensive medications (untreated hypertensive) and thus was eligible for the simulation study. Participant A’s BP (systolic blood pressure 153/diastolic blood pressure 93) was measured without antihypertensive treatment (measured untreated BP). Participant A was matched to a treated hypertensive participant with similar age, sex, race and body mass index (Participant X). Participant X’s BP (150/90) was used as the simulated treated BP (BP that would have been measured for Participant A had he/she been taking antihypertensive medications). Using the simulated treated BP, the following imputation methods were conducted: #1, set as missing; #2, single constant addition (systolic BP 10/diastolic BP 5); #3, class-specific constant addition; #4, truncated normal regression; #5, truncated normal regression with prior visit BP and antihypertensive treatment. (TIF) [file pone.0179234.s001.tif]

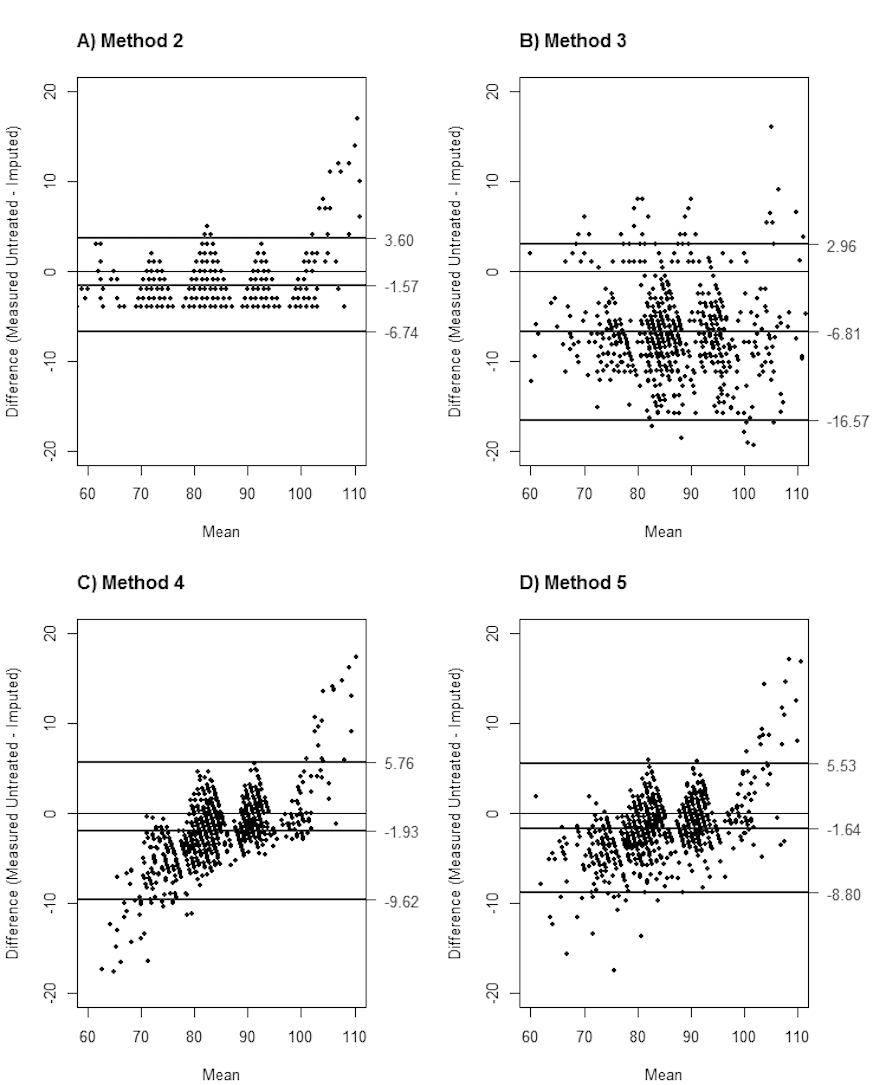

Supplement: S2 Fig — The measured untreated diastolic blood pressure (DBP) was treated as gold standard for the Bland-Altman plots with confidence intervals at 2.5%-97.5%. Imputed underlying DBP was derived using methods 2–5 (A-D). (TIF) [file pone.0179234.s002.tif]
