# Supplementary material for: Methods to estimate underlying blood pressure: The Atherosclerosis Risk in Communities (ARIC) Study
Source: PLoS One. 2017 Jul 11;12(7):e0179234. doi: 10.1371/journal.pone.0179234 (PMC5507409; doi:10.1371/journal.pone.0179234)
Supplement: S1 Table — Adapted from Wu J, Kraja AT, Oberman A, Lewis CE, Ellison RC, Arnett DK, et al. A summary of the effects of antihypertensive medications on measured blood pressure. Am J Hypertens 2005 Jul;18(7):935–942. The expected effect of each medication class was estimated and listed overall and by race for ACE inhibitors. If the medication was used as part of combination therapy, the average effects of the non-primary medication were reduced. This weighted effect was expected to be influenced by whether a diuretic was part of the combination therapy. Abbreviations: BP, blood pressure; SBP, systolic blood pressure; DBP, diastolic blood pressure; ACE, angiotensin-converting enzyme. (DOCX) [file pone.0179234.s003.docx]

| Medication | Average Monotherapy Effect (SBP/DBP mmHg) | Weighted Effect for Combination Therapy (SBP/DBP %) | |
| --- | --- | --- | --- |
|  |  | **Diuretic** | **No Diuretic** |
| ACE‡ inhibitor, African American | 6.8/6.6 | 29/29 | 50/41 |
| ACE‡ inhibitor,  European American | 14.3/10.4 | 47/56 | 50/42 |
| Alpha blocker | 15.5/11.7 | 11/34 | 48/50 |
| Beta blocker | 14.8/12.2 | 11/34 | 48/50 |
| Calcium channel blocker | 15.3/10.5 | 11/34 | 48/50 |
| Diuretic | 15.5/9.0 | 11/34 | 48/50 |
| Miscellaneous | 14.8/10.5 | 11/34 | 48/50 |
