# Supplementary material for: Methods to estimate underlying blood pressure: The Atherosclerosis Risk in Communities (ARIC) Study
Source: PLoS One. 2017 Jul 11;12(7):e0179234. doi: 10.1371/journal.pone.0179234 (PMC5507409; doi:10.1371/journal.pone.0179234)
Supplement: S2 Table — At each visit, participants were included in the analysis if they did not have missing age, sex, race, body mass index (BMI), antihypertensive medication use and antihypertensive medication class. Participants were further excluded if they had blood pressure (BP) measured at only 1 visit. Of the remaining participants, they were classified according to medication status (treated hypertensive, untreated) and BP levels among untreated (non-hypertensive, untreated hypertensive). Untreated hypertensive participants were included in the simulation study if they had at least 1 match among the treated hypertensive according to age, sex, race and BMI. (DOCX) [file pone.0179234.s004.docx]

|  | Visit 1 | Visit 2 | Visit 3 | Visit 4 | Visit 5 |
| --- | --- | --- | --- | --- | --- |
| Total | 15,792 | 14,348 | 12,887 | 11,656 | 6,538 |
| Without Missing Data | 14,644 | 14,275 | 12,826 | 11,585 | 6,221 |
| Treated Hypertensive | 4,361 | 4,720 | 4,864 | 5,079 | 4,692 |
| Untreated | 10,283 | 9,555 | 7,962 | 6,506 | 1,529 |
| Non-hypertensive | 9,122 | 8,477 | 6,923 | 5,432 | 1,177 |
| Untreated Hypertensive | 1,161 | 1,078 | 1,039 | 1,074 | 352 |
| Matched Untreated Hypertensive | 1,161 | 1,078 | 1,012 | 1,025 | 333 |
