# Supplementary material for: Methods to estimate underlying blood pressure: The Atherosclerosis Risk in Communities (ARIC) Study
Source: PLoS One. 2017 Jul 11;12(7):e0179234. doi: 10.1371/journal.pone.0179234 (PMC5507409; doi:10.1371/journal.pone.0179234)
Supplement: S3 Table — Abbreviations: BMI, body mass index; CHD, coronary heart disease; SD, standard deviation. (DOCX) [file pone.0179234.s005.docx]

|  | Untreated Hypertensive Participants | Treated Hypertensive Participants | P-value |
| --- | --- | --- | --- |
| Sample size | 1,161 | 4,361 |  |
| Mean age, yrs (SD) | 55.2 (5.7) | 55.4 (5.7) | 0.30 |
| Male (%) | 606 (52.2) | 1,716 (39.3) | <0.01 |
| African American (%) | 487 (41.9) | 1,627 (37.3) | <0.01 |
| Mean BMI, kg/m^2^ (SD) | 55.2 (5.7) | 55.4 (5.7) | 0.30 |
| Center (%) |  |  | <0.01 |
| Forsythe | 237 (20.4) | 921 (21.1) |  |
| Jackson | 450 (38.8) | 1,439 (33.0) |  |
| Minneapolis | 252 (21.7) | 864 (19.8) |  |
| Washington | 222 (19.1) | 1,137 (26.1) |  |
| Education less than  high school (%) | 821 (70.8) | 3,041 (69.8) | 0.56 |
| Current smokers (%) | 277 (23.9) | 994 (22.8) | 0.47 |
| Current drinkers (%) | 645 (55.9) | 2,055 (47.3) | <0.01 |
| Kidney dysfunction (%) | 12 (1.0) | 111 (2.6) | <0.01 |
| Diabetes (%) | 143 (12.5) | 877 (20.4) | <0.01 |
| Prevalent CHD (%) | 21 (1.9) | 455 (10.7) | <0.01 |
| Prevalent heart failure (%) | 3 (0.3) | 608 (14.1) | <0.01 |
| Parental history of CHD (%) | 77 (8.3) | 433 (12.3) | <0.01 |
