# Supplementary material for: Methods to estimate underlying blood pressure: The Atherosclerosis Risk in Communities (ARIC) Study
Source: PLoS One. 2017 Jul 11;12(7):e0179234. doi: 10.1371/journal.pone.0179234 (PMC5507409; doi:10.1371/journal.pone.0179234)
Supplement: S4 Table — Abbreviations: BMI, body mass index; CHD, coronary heart disease; SD, standard deviation. (DOCX) [file pone.0179234.s006.docx]

|  | Untreated Hypertensive Participants | Treated Hypertensive Participants | P-value |
| --- | --- | --- | --- |
| Sample size | 1,012 | 4,864 |  |
| Mean age, yrs (SD) | 61.2 (5.8) | 61.0 (5.7) | 0.26 |
| Male (%) | 493 (48.7) | 2,083 (42.8) | <0.01 |
| African American (%) | 276 (27.3) | 1,580 (32.5) | <0.01 |
| Mean BMI, kg/m^2^ (SD) | 61.2 (5.8) | 61.0 (5.7) | 0.26 |
| Center (%) |  |  | <0.01 |
| Forsythe | 255 (25.2) | 1,092 (22.5) |  |
| Jackson | 246 (24.3) | 1,379 (28.4) |  |
| Minneapolis | 262 (25.9) | 1,067 (21.9) |  |
| Washington | 249 (24.6) | 1,326 (27.3) |  |
| Education less than  high school (%) | 780 (77.2) | 3,588 (73.9) | 0.03 |
| Current smokers (%) | 183 (18.2) | 739 (15.3) | 0.03 |
| Current drinkers (%) | 539 (53.5) | 2,126 (43.9) | <0.01 |
| Diabetes (%) | 134 (13.3) | 1,208 (25.0) | <0.01 |
| Prevalent CHD (%) | 41 (4.1) | 691 (14.4) | <0.01 |
| Prevalent heart failure (%) | 11 (1.1) | 447 (9.3) | <0.01 |
| Parental history of CHD (%) | 75 (8.8) | 479 (12.0) | <0.01 |
