# Supplementary material for: Methods to estimate underlying blood pressure: The Atherosclerosis Risk in Communities (ARIC) Study
Source: PLoS One. 2017 Jul 11;12(7):e0179234. doi: 10.1371/journal.pone.0179234 (PMC5507409; doi:10.1371/journal.pone.0179234)
Supplement: S5 Table — Abbreviations: BMI, body mass index; CHD, coronary heart disease; SD, standard deviation. (DOCX) [file pone.0179234.s007.docx]

|  | Untreated Hypertensive Participants | Treated Hypertensive Participants | P-value |
| --- | --- | --- | --- |
| Sample size | 1,025 | 5,079 |  |
| Mean age, yrs (SD) | 64.0 (5.6) | 63.7 (5.7) | 0.19 |
| Male (%) | 438 (42.7) | 2,226 (43.8) | 0.54 |
| African American (%) | 253 (24.7) | 1,564 (30.8) | <0.01 |
| Mean BMI, kg/m^2^ (SD) | 64.0 (5.6) | 63.7 (5.7) | 0.19 |
| Center (%) |  |  | <0.01 |
| Forsythe | 210 (20.5) | 1,102 (21.7) |  |
| Jackson | 231 (22.5) | 1,392 (27.4) |  |
| Minneapolis | 303 (29.6) | 1,152 (22.7) |  |
| Washington | 281 (27.4) | 1,433 (28.2) |  |
| Education less than  high school (%) | 833 (81.3) | 3,833 (75.6) | <0.01 |
| Current smokers (%) | 155 (15.2) | 654 (13.0) | 0.07 |
| Current drinkers (%) | 542 (53.2) | 2,103 (41.9) | <0.01 |
| Kidney dysfunction (%) | 36 (3.5) | 551 (10.9) | <0.01 |
| Diabetes (%) | 128 (12.7) | 1,294 (25.8) | <0.01 |
| Prevalent CHD (%) | 32 (3.1) | 762 (15.3) | <0.01 |
| Prevalent heart failure (%) | 13 (1.3) | 354 (7.1) | <0.01 |
| Parental history of CHD (%) | 89 (10.3) | 490 (11.7) | 0.26 |
