# Supplementary material for: Methods to estimate underlying blood pressure: The Atherosclerosis Risk in Communities (ARIC) Study
Source: PLoS One. 2017 Jul 11;12(7):e0179234. doi: 10.1371/journal.pone.0179234 (PMC5507409; doi:10.1371/journal.pone.0179234)
Supplement: S6 Table — Abbreviations: BMI, body mass index; CHD, coronary heart disease; SD, standard deviation. (DOCX) [file pone.0179234.s008.docx]

|  | Untreated Hypertensive Participants | Treated Hypertensive Participants | P-value |
| --- | --- | --- | --- |
| Sample size | 333 | 4,692 |  |
| Mean age, yrs (SD) | 75.9 (5.3) | 75.9 (5.2) | 0.91 |
| Male (%) | 129 (38.7) | 1,966 (41.9) | 0.28 |
| African American (%) | 44 (13.2) | 1,313 (28.0) | <0.01 |
| Mean BMI, kg/m^2^ (SD) | 75.9 (5.3) | 75.9 (5.2) | 0.91 |
| Center (%) |  |  | <0.01 |
| Forsythe | 89 (26.7) | 954 (20.3) |  |
| Jackson | 39 (11.7) | 1,210 (25.8) |  |
| Minneapolis | 129 (38.7) | 1,263 (26.9) |  |
| Washington | 76 (22.8) | 1,265 (27.0) |  |
| Education less than  high school (%) | 300 (90.4) | 3,907 (83.4) | <0.01 |
| Current smokers (%) | 23 (7.1) | 251 (5.6) | 0.3 |
| Current drinkers (%) | 200 (62.1) | 2,054 (45.7) | <0.01 |
| Kidney dysfunction (%) | 59 (17.9) | 1,541 (33.2) | <0.01 |
| Diabetes (%) | 44 (13.6) | 1,810 (39.5) | <0.01 |
| Prevalent CHD (%) | 10 (3.1) | 855 (18.5) | <0.01 |
| Prevalent heart failure (%) | 0 (0.0) | 143 (3.1) | <0.01 |
| Parental history of CHD (%) | 17 (6.1) | 442 (11.1) | 0.01 |
