# Supplementary material for: Methods to estimate underlying blood pressure: The Atherosclerosis Risk in Communities (ARIC) Study
Source: PLoS One. 2017 Jul 11;12(7):e0179234. doi: 10.1371/journal.pone.0179234 (PMC5507409; doi:10.1371/journal.pone.0179234)
Supplement: S7 Table — The relative bias (%) was calculated as the average relative difference in regression coefficient from each simulation (100 x [βimputation method−βmeasured untreated BP] / βmeasured untreated BP). Controls were matched on diabetes and kidney dysfunction status. Abbreviations: SBP, systolic blood pressure; Var, variance; DBP, diastolic blood pressure. (DOCX) [file pone.0179234.s009.docx]

|  |  | Measured | Method 1 | Method 2 | Method 3 | Method 4 | Method 5 | |
| --- | --- | --- | --- | --- | --- | --- | --- | --- |
| SBP | | | | | | | |  |
| Intercept | Beta (Var) | 114 | 105.69 | 113.61 (5.50e-4) | 113.90 (8.30e-4) | 113.40 (4.30e-4) | 113.46 (3.40e-4) | |
|  | Relative bias |  | 7.29 | 0.34 | 0.09 | 0.53 | 0.47 | |
| Age | Beta (Var) | 0.86 | 0.34 | 0.81 (8.90e-6) | 0.86 (1.40e-5) | 0.82 (6.90e-6) | 0.83 (6.30e-6) | |
|  | Relative bias |  | 60.47 | 5.82 | 0.00 | 4.65 | 3.49 | |
| DBP | | | | | | | |  |
| Intercept | Beta (Var) | 71.39 | 87.64 | 71.56 (7.00e-4) | 72.04 (0.02) | 71.43 (5.20e-4) | 71.43 (1.50e-4) | |
|  | Relative bias |  | 22.76 | 0.24 | 0.91 | 0.06 | 0.06 | |
| Age before 60 yrs | Beta (Var) | 0.03 | 0.59 | 0.04 (1.90e-6) | 0.07 (3.90e-6) | 0.04 (1.00e-6) | 0.03 (1.30e-6) | |
|  | Relative bias |  | 1866.67 | 33. 33 | 133.33 | 33.33 | 33.33 | |
| Age after 60 yrs | Beta (Var) | -0.11 | -0.74 | -0.09 (7.20e-6) | -0.01 (3.40e-5) | -0.05 (7.20e-6) | -0.06 (3.80e-6) | |
|  | Relative bias |  | -572.73 | 18.18 | 90.91 | 54.55 | 45.45 | |
